# Between Fate Choice and Self-Renewal—Heterogeneity of Adult Neural Crest-Derived Stem Cells

**DOI:** 10.3389/fcell.2021.662754

**Published:** 2021-04-08

**Authors:** Anna L. Höving, Beatrice A. Windmöller, Cornelius Knabbe, Barbara Kaltschmidt, Christian Kaltschmidt, Johannes F. W. Greiner

**Affiliations:** ^1^Department of Cell Biology, University of Bielefeld, Bielefeld, Germany; ^2^Institute for Laboratory- and Transfusion Medicine, Heart and Diabetes Centre North Rhine-Westphalia (NRW), Ruhr University Bochum, Bad Oeynhausen, Germany; ^3^Forschungsverbund BioMedizin Bielefeld FBMB e.V., Bielefeld, Germany; ^4^Molecular Neurobiology, University of Bielefeld, Bielefeld, Germany

**Keywords:** adult stem cells, neural crest-derived stem cells, heterogeneity, single cell analysis, fate choice, neural crest

## Abstract

Stem cells of the neural crest (NC) vitally participate to embryonic development, but also remain in distinct niches as quiescent neural crest-derived stem cell (NCSC) pools into adulthood. Although NCSC-populations share a high capacity for self-renewal and differentiation resulting in promising preclinical applications within the last two decades, inter- and intrapopulational differences exist in terms of their expression signatures and regenerative capability. Differentiation and self-renewal of stem cells in developmental and regenerative contexts are partially regulated by the niche or culture condition and further influenced by single cell decision processes, making cell-to-cell variation and heterogeneity critical for understanding adult stem cell populations. The present review summarizes current knowledge of the cellular heterogeneity within NCSC-populations located in distinct craniofacial and trunk niches including the nasal cavity, olfactory bulb, oral tissues or skin. We shed light on the impact of intrapopulational heterogeneity on fate specifications and plasticity of NCSCs in their niches *in vivo* as well as during *in vitro* culture. We further discuss underlying molecular regulators determining fate specifications of NCSCs, suggesting a regulatory network including NF-κB and NC-related transcription factors like SLUG and SOX9 accompanied by Wnt- and MAPK-signaling to orchestrate NCSC stemness and differentiation. In summary, adult NCSCs show a broad heterogeneity on the level of the donor and the donors’ sex, the cell population and the single stem cell directly impacting their differentiation capability and fate choices *in vivo* and *in vitro*. The findings discussed here emphasize heterogeneity of NCSCs as a crucial parameter for understanding their role in tissue homeostasis and regeneration and for improving their applicability in regenerative medicine.

## Introduction: Intrapopulational Heterogeneity of Adult Stem Cells

**FIGURE 1:**
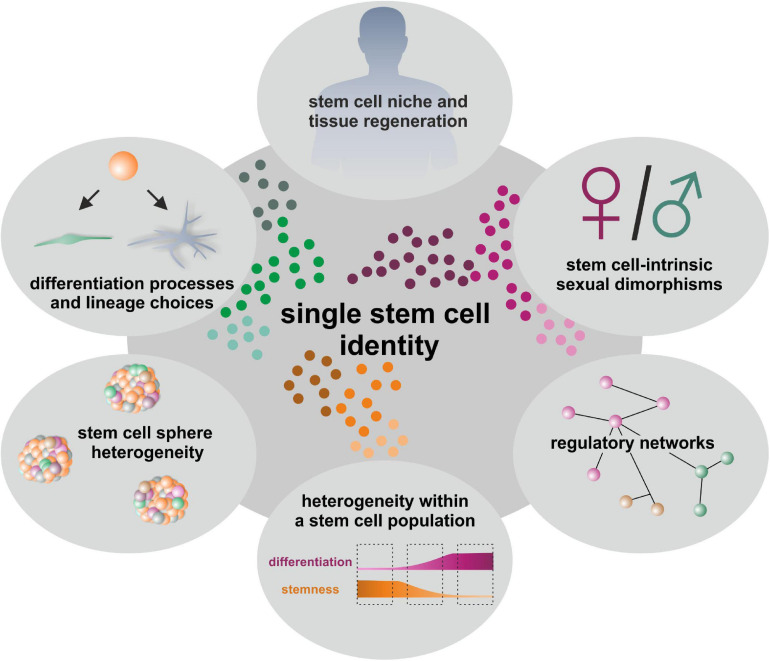
Determination of single stem cell identities gives insights into linage choices, regulatory networks, niches and model systems like cultured stem cell-derived spheroids as well as tissue regeneration.

Adult stem cells (ASCs) are undifferentiated cells capable of self-renewal as well as multi-lineage differentiation and are located in a broad range of niches throughout the human body (Rochat et al., 1994; Johansson et al., 1999; Pittenger et al., 1999; Messina et al., 2004; Hauser et al., 2012; Höving et al., 2020b). By giving rise to a variety of specialized cell types, ASCs not only participate to normal adult tissue homeostasis and endogenous regeneration processes upon injury or inflammation, but also harbor an enormous potential for applications in regenerative medicine. Differentiation and self-renewal of stem cells in developmental and regenerative contexts are partially regulated by the niche or culture condition and further influenced by single cell decision processes. Cell-to-cell variation and heterogeneity can thus be considered as fundamental and intrinsic characteristics of adult stem cell populations. Utilization of bulk samples thus not only limits the degree of resolution of cell type definitions but can also mask crucial information about subcellular heterogeneity within stem cell populations and their lineage choices (Moignard and Gottgens, 2016; Griffiths et al., 2018). In this regard, first observations of heterogeneity within a defined cell population were reported by Fleming and colleagues. In this pioneering study, the authors described functional heterogeneity within a phenotypically defined Thy1.1^lo^/lin^–^/Sca-1^+^ multipotent hematopoietic stem cell (HSC) population which was associated with the cell cycle status. In detail, murine Thy1.1^lo^/lin^–^/Sca-1^+^ cells in the G0/G1 phase showed significantly higher capacities for radioprotection and long-term multilineage reconstitution of peripheral blood after injection into lethally irradiated recipient mice compared to Thy1.1^lo^/lin^–^/Sca-1^+^ cells in the S/G2/M phase (Fleming et al., 1993). Following this pioneering work, single cell technologies have been developed rapidly in recent years to dissect cellular heterogeneity and identify subpopulations even within a “homogenous” stem cell population, providing deeper insight into their behavior and biological function (Fletcher et al., 2017; Gadye et al., 2017; Joost et al., 2018). For HSCs, recent single cell observations ranged from reporting heterogeneity of HSCs within the bone marrow (Moignard et al., 2013; Yu et al., 2016), for instance regarding the cell-cycle status of pre-HSCs (Zhou et al., 2016), up to providing the regulatory landscape of human hematopoietic differentiation on single cell level (Buenrostro et al., 2018). For long time, transplantation assays did not reveal functional differences but the observed diversity at transcriptional and epigenetic level may provide information to engineer HSCs for clinical applications and emphasize the importance of single cell measurements (Yu et al., 2016; Zhou et al., 2016; Buenrostro et al., 2018). Very recently, Rodriguez-Fraticelli and colleagues combined single cell transcriptome analysis with functional transplantation assays and discovered the transcription factor Tcf15 to be crucial for the regulation of HSC long-term regenerative capacity *in vivo* (Rodriguez-Fraticelli et al., 2020). Next to well-studied HSCs, single cell analysis was successfully applied to investigate the differentiation dynamics of primary human skeletal muscle myoblasts (Trapnell et al., 2014) and neural stem cells (NSCs). Here, Suslov et al. (2002) as well as Dulken et al. (2017) reported intra-clonal heterogeneity and cell-lineage diversity in NSCs cultured as neurospheres, initiating a discussion regarding their applicability as a model system. Mesenchymal stem cells (MSCs) were likewise observed to show not only functional and molecular differences between clones, but also intra-clonal heterogeneity and cell-to-cell variations (reviewed in McLeod and Mauck, 2017). For instance, Freeman et al. (2015) utilized single cell RNA-sequencing (scRNA-seq) to demonstrate unique profiles of lineage priming in individual bone marrow-derived MSC. Although multipotency-associated transcriptional profiles were consistently observed, single MSCs showed varying levels of genes associated with differentiation and immunomodulation, which could not be ascribed to proliferation state or other cellular processes (Freeman et al., 2015). The possibility to define intercellular heterogeneity via single cell analysis becomes thus more and more evident to understand cell-to-cell variations within a tissue or a distinct population of cells and stem cells. Determining the identities of ASCs at single cell resolution will improve our insights into lineage choices and underlying regulatory networks in endogenous tissue regeneration as well as in the context of cell-intrinsic sexual dimorphisms (Figure 1). In addition, the systematic investigation of cellular heterogeneity in ASC-based model systems like cultured stem cell-derived spheroids may broaden the applicability of ASCs in drug development and regenerative medicine (Figure 1).

Among the broad range of ASCs in their diverse niches, adult neural crest-derived stem cells (NCSCs) reveal a particularly broad differentiation potential (see also section “Adult Neural Crest-Derived Stem Cells—From Individual Developmental Drivers to Mediators of Regeneration During Adulthood”) and are therefore highly promising candidates for studying heterogeneity of stem cell populations and fate decisions. This review will summarize current tools for analyzing gene expression profiles of single (neural crest-derived) stem cells via scRNA-seq and discuss recent observations of single cell heterogeneity in mammalian NCSC-populations located in distinct craniofacial and trunk niches including the nasal cavity, olfactory bulb, carotid body, cornea of the eye, oral tissues, craniofacial and trunk skin, dorsal root ganglia, sciatic nerve, bone marrow, heart and gut. We will further assess underlying molecular regulators orchestrating fate specifications and stemness of NCSCs.

## Technical Excursion: State of the Art Tools and Challenges of Analyzing Single Stem Cells

**FIGURE 2:**
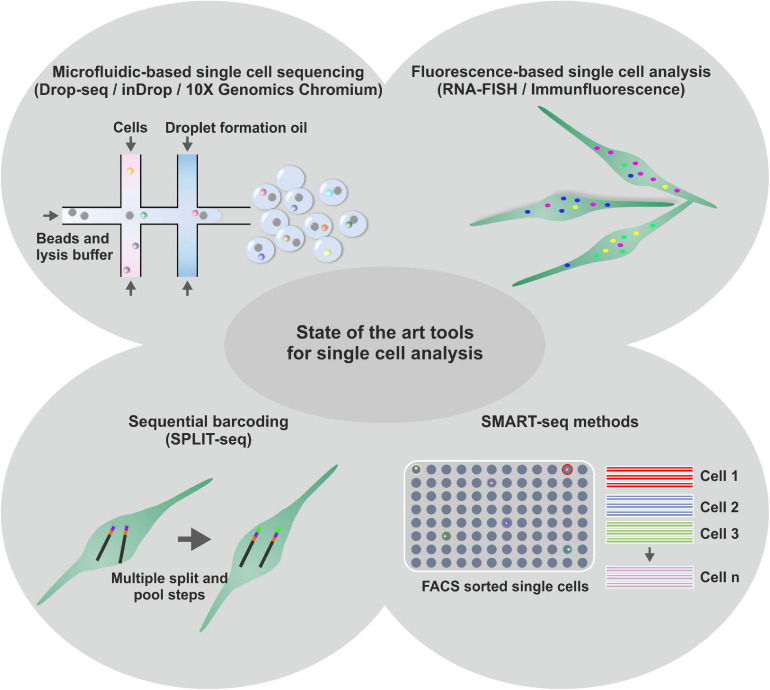
Schematic representation of diverse state-of-the-art tools for single cell analysis. Next to microfluidic based single cell sequencing methods, like Drop-seq, inDrop or 10X Genomic Chromium, for high throughput transcriptome analysis of single cells, fluorescence-based methods are used to investigate single cell transcriptomes. Further, Sequential barcoding methods like SPLIT-seq are applied for high throughput single cell sequencing. Less throughput, but higher precision was shown to be achieved using SMART-Seq methods with prior fluorescence activated cell sorting (FACS).

The major challenge in performing single stem cell experiments lies in the extremely low amount of starting material that can be isolated from one single cell. This little starting material can lead to a loss of information in the resulting cDNA libraries as well as to a phenomenon called drop-out, if a gene is expressed in a cell but not detected in RT-PCR or scRNA-seq (Kumar et al., 2017). On the contrary, fluctuations in the transcription rates of a gene (termed as transcriptional noise) occur based on random distributions of extremely low intracellular concentrations of the acting molecules (McAdams and Arkin, 1999; Kumar et al., 2017). To gain a deeper insight into cellular heterogeneity, the analysis of a large quantity of single cells is necessary making high throughput methods inevitable. Next to appropriate sample acquisition (reviewed in Saliba et al., 2014), various strategies and platforms for analyzing gene expression of single stem cells have been established in the last years (see Figure 2). As an image-based method, RNA-fluorescence *in situ* hybridization (FISH) utilizes fluorescently labeled oligonucleotides that hybridize to their complementary counterparts in a fixed cell. Thus, mRNA molecules can be visualized as diffraction limited spots providing information about their spatial distribution within the cell. However, the potential number of simultaneously assayed genes is restricted by the availability of microscope filters but can be increased to a certain extend by combinatorial and sequential barcoding (Lubeck and Cai, 2012; Lubeck et al., 2014; Moffitt et al., 2016). For in-depth analyses, global gene expression profiling by scRNA-seq harbors the potential to discriminate thousands of transcripts (biomarkers) and their expression levels. One first scRNA-seq technique was SMARTseq/SMARTseq2 (switching mechanism at 5′ end of the RNA transcript), which enables the generation of full-length cDNA transcripts of one single cell (Ramsköld et al., 2012; Picelli et al., 2014). However, prior to the application of SMARTseq methods, single cells have to be prepared by either FACS or single cell dilution limiting the amount of analyzed single cells. Rosenberg and colleagues developed spilt-pool ligation-based transcriptome sequencing (SPLiT-seq), which applies four rounds of combinatorial barcoding to a suspension of formaldehyde-fixed cells (Rosenberg et al., 2018). Sequential barcoding of the mRNA results in more than 21 million of possible barcode combinations starting from 96 individual cells enabling high-grade multiplexing in a single sequencing run and reducing costs to 0.1 USD per cell. However, the workflow prior to sequencing requires a range of pipetting steps, which is time-consuming and limits the number of simultaneously analyzed cells. Microfluidic-based systems are working with nanoliter-scale volumes allowing substantial reduction of the rate of external contaminations (Blainey and Quake, 2011). Additionally, the small volumes avoid an excessive dilution of the input RNA as well as concomitant potential bias from pre-amplification steps and technical variability (Wu et al., 2014). Linking microfluidic systems and droplet barcoding strategies currently represents the leading edge of single cell research (Klein et al., 2015; Macosko et al., 2015; Zilionis et al., 2016; Zheng et al., 2017). Here, individual (stem) cells are trapped in a nanoliter-scale droplet containing barcoding oligonucleotide primers as well as reverse transcriptase (RT)- and lysis reagents. Inside the droplet, a single cell is lysed followed by mRNA-barcoding during RT-reaction. Barcoded single cell mRNAs are pooled and prepared cDNA libraries can be processed for sequencing. Due to the initial barcoding, the information derived from a pool of cells can be separated *in silico* to create expression profiles of the corresponding cells (Klein et al., 2015; Zilionis et al., 2016; Zheng et al., 2017). With regard to nanoliter-scale working-volumes and pooling of labeled single cell mRNA, the linkage of microfluidic systems and droplet barcoding strategies allows a significant decrease in the respective amount of required reagents and therefore a dramatic reduction of costs per sequenced cell (Klein et al., 2015; Ziegenhain et al., 2017). Currently, the platforms Drop-seq (Macosko et al., 2015), inDrop (Klein et al., 2015; Zilionis et al., 2016), and 10X Genomics Chromium (Zheng et al., 2017) are most widely applied for microfluidic-based generation of single cell libraries followed by scRNA-seq. In a recent side-by-side comparison by Zhang et al. (2019), all systems were shown to be satisfactory efficient in detection of transcripts. The authors concluded 10X to be a reasonable and safe option for a wide range of applications, while Drop-seq being more cost-efficient for abundant samples and inDrop being the best choice for detecting low-abundance transcripts or for establishing custom protocols (Zhang et al., 2019). Although the sorting of a cell population of interest prior to sequencing is not supported and the analysis of distinct small cell populations is thus exacerbated, microfluidic systems are well-suited to address the challenge of scRNA-seq of stem cells in a cost-efficient manner. Regarding the techniques introduced above, the following chapters will discuss single cell data reporting heterogeneity in NCSC populations and underlying regulatory networks.

## Adult Neural Crest-Derived Stem Cells—From Individual Developmental Drivers to Mediators of Regeneration During Adulthood

**FIGURE 3:**
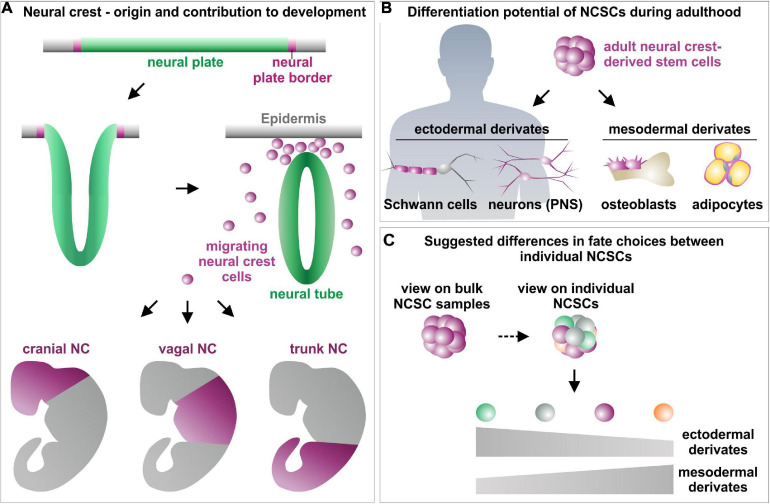
Schematic view on the role of neural crest stem cells in embryogenesis **(A)** and adulthood **(B)** as well as suggested differences between favored fate choices of single NCSCs **(C)**. **(A)** Modified from Greiner et al. (2019a) and Srinivasan and Toh (2019), NC, Neural crest; NCSCs, Neural crest-derived stem cells; PNS, Peripheral nervous system.

The neural crest (NC) was described the first time by Wilhelm His in 1868 as the intermediate chord (“Zwischenstrang”) between the neural tube and the future ectoderm in the developing chick embryo (His, 1868). During neurulation, premigratory neural crest cells arise from the neural plate border and undergo epithelial to mesenchymal transition (EMT) after formation of the neural tube (Figure 3A). Following EMT, neural crest cells migrate out of their niche and give rise to a broad variety of ectodermal and mesenchymal cell types, thereby fundamentally contributing to embryonic development (Dupin and Sommer, 2012; Kaltschmidt et al., 2012; Etchevers et al., 2019). According to their position on the anteroposterior axis, migration behavior and differentiation potential, migrating NC cells are divided into cranial, vagal (including cardiac), trunk and sacral NC cells (Yntema and Hammond, 1954; Le Douarin and Teillet, 1974; Noden, 1975, 1978a; reviewed by Rothstein et al., 2018). Cranial NC cells firstly undergo EMT and form a range of craniofacial cell types and tissues such as peripheral nerves, melanocytes, thyroid cells, teeth and most of the craniofacial skeleton (Johnston, 1966; Noden, 1978a, b; Baker et al., 1997; see reviews from Graham et al., 2004; Kaltschmidt et al., 2012; Rocha et al., 2020 for overview; Figure 3A). Vagal NC cells give rise to enteric neurons and glia forming the enteric nervous system of the foregut and stomach, while an anterior localized subset, the cardiac NC cells, contributes to septation of the outflow tract in the developing heart (Phillips et al., 1987; Burns and Douarin, 1998). Trunk NC cells were reported to differentiate into sensory and sympathetic ganglia, melanocytes and the adrenal medulla (Figure 3A). Sacral NC cells, which have only been identified in amniotes so far, colonize the gut after their vagal counterparts to form enteric glia and neurons innervating the hindgut (Yntema and Hammond, 1954; Le Douarin and Teillet, 1973; Epstein et al., 1994; see review from Rothstein et al., 2018 for overview). Notably, NC cells not only give rise to distinct cell types in the developing tissues, but even guide patterning and differentiation of their target tissues during embryogenesis (Rios et al., 2011; Faure et al., 2015). For instance, in the avian system, the cephalic neural crest (CNC) has been shown to control development of the forebrain and midbrain. Here, the CNC regulates the shaping and size of the pre-otic brain by the expression of bone morphogenetic protein (BMP) antagonists like Gremlin and Noggin (Creuzet, 2009). Accordingly, mutations in key transcription factors regulating migration and differentiation of NC cells, like SLUG, SOX10, SNAIL, or TWIST lead to NC maldevelopment and specific disease phenotypes. Amongst others, these so-called neurocristopathies (Bolande, 1974) particularly include tumors as melanoma, neuroblastoma or neurofibroma, malformations like cleft lip or palate, heart malformations and craniofacial defects (Kabuki syndrome) as well as Hirschsprung’s disease, Waardenburg syndrome or Charcot–Marie–Tooth disease (Bolande, 1974; Wilkie and Morriss-Kay, 2001; Amiel et al., 2008; Vega-Lopez et al., 2018; Greiner et al., 2019b). Interestingly, certain neurocristopathies like Hirschsprung’s disease, Waardenburg syndrome, melanoma or meningioma were reported to occur in a sex-specific manner (reviewed in Greiner et al., 2019b). While Hirschsprung’s disease, Waardenburg syndrome and melanoma were described to have a prevalence in males (Amiel et al., 2008; Karimkhani et al., 2017), meningiomas occur twice as often in female individuals (Korhonen et al., 2006), emphasizing the potential heterogeneity between embryonic NC populations of distinct sexes (reviewed in Greiner et al., 2019b). In addition, Lignell et al. (2017) revealed heterogeneity even within embryonic NC stem cell populations in the chick dorsal neural tube by demonstrating the presence of distinct premigratory and early migratory neural crest populations in the developing dorsal midbrain. These observations emphasize the heterogeneity of cells located in the neural crest stem cell niche and provide hints for spatially and transcriptionally distinct subpopulations at single cell resolution (Lignell et al., 2017).

Beyond their tremendous role in embryogenesis, neural crest stem cells also remain in distinct niches as quiescent stem cell pools into adulthood (Sieber-Blum and Grim, 2004; Widera et al., 2007; Dupin and Sommer, 2012; Hauser et al., 2012). These NCSCs can be found in various tissues of the adult human body, for example the bone marrow (Coste et al., 2017), skin (Toma et al., 2001), heart (Höving et al., 2020b), cornea (Katikireddy et al., 2016), hair follicles (Clewes et al., 2011), dental pulp (Waddington et al., 2009), palatum (Widera et al., 2009), or the respiratory and olfactory epithelium of the nasal cavity (Murrell et al., 2008; Hauser et al., 2012; Schurmann et al., 2017). Adult NCSCs are known to possess a high capacity for self-renewal as well as an extended multipotency, revealed by an extraordinary high differentiation potential particularly into mesenchymal and ectodermal cell types *in vitro* and *in vivo* (Figure 3B; Murrell et al., 2008; Shakhova and Sommer, 2008; Müller et al., 2015; Hofemeier et al., 2016; Greiner et al., 2019a). Common characteristics of NCSCs further include expression of the intermediate filament Nestin and NC-associated markers like SOX9, SOX10, SLUG, SNAIL, or TWIST as well as the capacity to form neurospheres *in vitro* (Nieto et al., 1994; Lothian and Lendahl, 1997; Southard-Smith et al., 1998; del Barrio and Nieto, 2002; Spokony et al., 2002; Kim et al., 2003; Soldatov et al., 2019). Their broad differentiation and migration capability and accessibility in the adult human organism make NCSCs promising candidates for pharmacological research (Matigian et al., 2010; Rodrigues et al., 2014; Müller et al., 2016) and regenerative medicine (Barnett et al., 2000; Tabakow et al., 2014; Müller et al., 2015). Here, NCSCs were reported to functionally recover animal models of Parkinson’s disease or spinal cord injury (Barnett et al., 2000; Murrell et al., 2008; Müller et al., 2015). Tabakow et al. (2014) impressively demonstrated the functional regeneration of supraspinal connections in a patient with transected spinal cord. Next to peripheral nerve bridging, the authors transplanted autologous olfactory ensheathing cells, a population of NCSCs located in the olfactory bulb, resulting in partial recovery of voluntary movement of the lower extremities (Tabakow et al., 2014).

Despite similarities in their clinical applicability and common characteristics, distinct populations of human NCSCs differ in their expression profiles and differentiation potential with particular regard to their tissue of origin (Höving et al., 2020b). In addition to differences between distinct populations of adult NCSCs, several very recent studies even point toward a cellular heterogeneity within respective NCSC-pools (Figure 3C; Young et al., 2016; see Table 1 for overview). The following chapters will focus on these heterogeneities in terms of NCSC-identities, fate decision and underlying regulatory networks.

**TABLE 1:** Overview of single cell experiments in NCSC populations.

NCSC population	Single cell method	Key findings	References
Murine horizontal basal cells (HBCs)	scRNA-seq using FACS and SMART-Seq technology/*in vivo* lineage tracing	• Direct fate conversion of quiescent HBCs into either sustentacular cells or globose basal cells prior to cell division • Olfactory neurogenesis via differentiation of globose basal cells • Canonical Wnt signaling regulates differentiation into the neuronal lineage	Fletcher et al., 2017
Murine HBCs	scRNA-seq using FACS and SMART-Seq technology/*in vivo* lineage tracing	• Injury activates a highly heterogenous transient state in HBCs • differentiation into sustentacular cells involved proliferation of HBCs	Gadye et al., 2017
Human HBCs from olfactory neuroepithelium	scRNA-seq using 10X Genomics Chromium	• Stemness state of HBCs under non-injury conditions suggested to be similar to injury conditions in rodents	Durante et al., 2020
Rodent olfactory ensheathing cells (OEC)	Single cell migration assays	• Distinct OEC subpopulations display different migratory properties	Huang et al., 2008
Human inferior turbinate stem cells (ITSCs)	Immunocytochemistry	• ITSC-derived neurons from female donors revealed elevated oxidative stress-induced cell death in comparison to male neurons	Ruiz-Perera et al., 2018
Human ITSCs	Immunocytochemistry	• ITSC clones revealed different ratios of ectodermal to mesodermal progeny upon differentiation	Greiner et al., 2011; Hauser et al., 2012
Murine Dental Pulp Stem/Progenitor Cells (DPSCs)	Immunocytochemistry	• DPSC clones reveal heterogeneity in neuronal differentiation capacity	Young et al., 2016
Human DPSCs	Immunocytochemistry and DNA microarray	• Human dental pulp stem cell clones reveal differences in proliferation, differentiation potential and gene expression, with characteristic DPSC-markers being mainly conserved	Kobayashi et al., 2020
Human DPSCs	FACS/Immunocytochemistry	• Canonical Wnt-GSK3β/β-catenin pathway contributes to DPSC differentiation into mature SMCs. However, different clones reveal heterogeneity in differentiation potential	Jiang et al., 2019
Human DPSCs	Single-cell Raman spectroscopy	• Subpopulations of highly proliferative/multipotent DPSCs and low proliferative/unipotent were identified in human third molars	Alraies et al., 2019
Murine hair follicle bulge stem cells	scRNa-seq using microfluidic based Fluidigm C1 Autoprep System	• Lack of common stemness expression signature but segregation of cell populations in relation to their location during tissue homeostasis	Joost et al., 2016
Murine hair follicle bulge stem cells	scRNa-seq using microfluidic based Fluidigm C1 Autoprep System/*in vivo* lineage tracing	• Stem cells activate interfollicular epidermis stem cell-like gene expression signature during wound healing even before migration to the lesion • great cellular plasticity of single stem cells allowing rapid transcriptional adaptations during wound healing	Joost et al., 2018
Embryonic quail mesencephalic neural crest cells	Analysis of single cell-derived clones	• Treatment with the morphogen Sonic Hedgehog (Shh) increased the number of multipotent subclones with the capacity to differentiate into glia, neurons, melanocytes, myofibroblasts and chondrocytes	Calloni et al., 2007, 2009
Embryonic quail mesencephalic neural crest cells	Analysis of single cell-derived clones	• Increased developmental potential into glial cells, neurons, melanocytes, smooth muscle cells, chondrocytes, and adipocytes after the simultaneous application of FGF8 and Shh	da Costa et al., 2018
Fetal rat sciatic nerve-derived NCSCs	FACS	• p75 + subpopulations were able to differentiate into neurons, Schwann cells and myofibroblasts while p75- cells were only able to differentiate into myofibroblasts	Morrison et al., 1999

## Fate Specifications and Heterogeneity of NCSCs From the Nasal Cavity and Olfactory Bulb

The mammalian nasal cavity and olfactory bulb (OB) are well-known to harbor distinct niches for adult NCSCs. Amongst others, these NCSC-populations include horizontal basal cells (HBCs) and olfactory ensheathing cells (OECs) residing in olfactory mucosa of the middle and superior turbinate (OECs and HBCs) and as well as the olfactory bulb (OECs) (Barnett et al., 2000; Murrell et al., 2008; Barraud et al., 2010; Suzuki et al., 2013; Tabakow et al., 2014). Further, olfactory ectomesenchymal stem cells (OE-MSC) within the lamina propria of the human olfactory mucosa were shown to express a range of neural as well as mesenchymal markers (Delorme et al., 2010). In addition, NCSCs were described to be located in the respiratory epithelium of the inferior turbinate (inferior turbinate stem cells, ITSCs) (Hauser et al., 2012) and in the lamina propria of middle turbinate tissue (middle turbinate stem cells, MTSCs) (Schurmann et al., 2017). Assessment of the heterogeneity of these stem cell populations is increasingly enabling promising insights into the control of fate decisions and lineage restrictions of mammalian stem cells and adult mammalian regeneration. In this regard, Fletcher et al. (2017) recently investigated a detailed map of lineage choices for HBCs in the murine postnatal olfactory epithelium using scRNA-seq combined with lineage tracing *in vivo*. Interestingly, an initial lineage trajectory of quiescent HBCs into either sustentacular cells via direct fate conversion or globose basal cells (GBCs) was reported to occur prior to cell division. For olfactory neurogenesis ensuring tissue homeostasis, GBCs proliferate and give rise to microvillous cells and olfactory sensory neurons, but also to Bowman’s gland. The authors further identified canonical Wnt-signaling as a major regulator driving the route of HBCs from quiescence to neuronal differentiation by promoting neuronal fate choices (Fletcher et al., 2017). Within the injured mouse olfactory epithelium, quiescent HBCs were shown to be activated and adopt a proliferative and transient state, which was found to be unique to injury (Gadye et al., 2017). Self-renewal of proliferating transient HBCs was reported to result in a resting HBC-population as well as in further enlargement of the transient HBC-population. Transient HBCs differentiated into the neuronal lineage via GBCs and microvillous cells toward mature olfactory sensory neurons with Sox2 being essential for transition from the activated to neuronal progenitor states. In contrast to non-injury conditions in tissue homeostasis (Fletcher et al., 2017), regeneration of sustentacular cells was demonstrated to involve proliferation of HBCs, although sustentacular cells likewise differentiated directly from the transient HBCs. Notably, activated single HBCs revealed a highly heterogeneous gene expression indicating a heterogeneity within the transient state of HBCs leading to designation of lineage commitment (Gadye et al., 2017). Using scRNA-seq of 28,726 single cells, Durante et al. (2020) very recently provided evidence for ongoing robust neurogenesis in the human olfactory epithelium under non-injury conditions contributing to sensing of smell. Next to a high ratio of immature to mature neurons, KRT5^+^/SOX2^+^ HBCs were frequently observed to possess a rounded, reactive-like morphology. The authors thus suggested the stemness state of HBCs in the OE of middle-aged humans to be similar to injury-induced epithelial reconstitution in rodents (Durante et al., 2020). Next to heterogeneity of NCSCs in the olfactory epithelium *in vivo*, Huang and colleagues reported differential migration behavior of distinct single murine OECs. Here, the distinct OEC-subpopulations defined the respective mode of migration as well as underlying distribution of the cytoskeleton (Huang et al., 2008).

**FIGURE 4:**
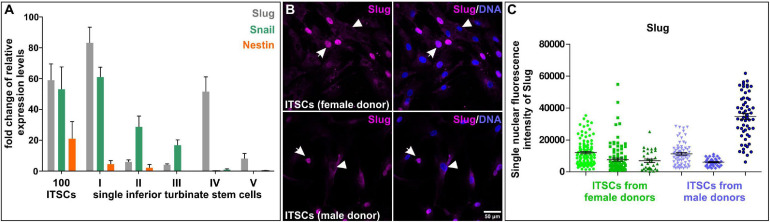
Heterogeneity of single neural crest-derived inferior turbinate stem cells (ITSCs) isolated from the human nasal cavity. **(A)** qPCR following SMART-Seq2 of single ITSCs showed heterogeneity in expression levels of Slug, Snail and Nestin. **(B,C)** Heterogeneous nuclear fluorescence intensities of Slug protein in single ITSCs.

Nearly a decade ago, we identified a population of NCSCs in the respiratory epithelium of the human inferior turbinate (Hauser et al., 2012). Inferior turbinate stem cells (ITSCs) could be propagated *in vitro* as free-floating neurospheres or within human blood plasma-derived 3D fibrin matrix. *In vitro* cultivated ITSCs showed the ability to self-renew and differentiate into mesenchymal derivates like adipocytes or osteoblasts, but also efficiently gave rise to glutamatergic and dopaminergic neurons (Greiner et al., 2011; Müller et al., 2015; Ruiz-Perera et al., 2018, 2020; Greiner et al., 2019a). Despite this commonly shared differentiation potential, ITSCs also revealed heterogeneities in their behavior during differentiation. In particular, we recently observed differences in neuroprotection of ITSC-derived glutamatergic neurons against oxidative stress in dependence on the sex of the stem cell donor. ITSC-derived neurons from female donors showed increased oxidative stress-induced neuronal death but also an elevated neuroprotection after stimulation of the transcription factor nuclear factor “kappa- light-chain-enhancer” of activated B cells (NF-κB) compared to their male counterparts (Ruiz-Perera et al., 2018). The observed differences in neuroprotection were accompanied by the presence of a sexually dimorphic protective gene expression program (Ruiz-Perera et al., 2018), suggesting further heterogeneities in differentiation behavior of ITSCs. In addition, we observed donor-dependent differences in the amount of p75^NTR^-positive stem cells in their niche within the inferior turbinate. Here, freshly dissociated tissue from individual donors showed 9.78% to even 26.50% of p75^NTR^-positive ITSCs depending on the donor. On single cell level, assessment of self-renewal in tertiary ITSC clones revealed different ratios of ITSC-derived ectodermal to mesenchymal progeny dependent on the distinct single stem cell (Greiner et al., 2011; Hauser et al., 2012), as schematically depicted in Figure 3C. Accordingly, recent qPCR-analysis following SMART-Seq2 of single self-renewing ITSCs emphasized the heterogeneity between individual stem cells on gene expression level. In particular, we observed great differences in expression levels of NCSC-markers SLUG, SNAIL and Nestin between single ITSCs (Figure 4A). Immunocytochemistry validated the heterogeneous amounts of nuclear localized SLUG protein, indicating variations in the presence and activity of SLUG depending on the individual stem cell state (Figure 4B, see also section “Molecular Regulators Defining Stemness and Fate Choices of NCSCs”). Since spontaneous differentiation of ITSCs was not observable under the applied culture conditions in our previous studies (Greiner et al., 2011; Hauser et al., 2012), we strongly suggest intrinsic differences between stem cell states to account for this heterogeneity. Interestingly, activation of SLUG is commonly noticed to regulate and determine stemness states of adult stem cells (Guo et al., 2012; Tang et al., 2016). The heterogeneous expression of major NCSC-markers like Nestin and regulatory transcription factors like SLUG may thus be directly associated to the observed differences in differentiation potentials between single ITSCs, as already described for NCSCs located in the oral cavity (Young et al., 2016, see below).

## Heterogeneity of NCSCs in the Oral Cavity

Next to the nasal cavity, NCSCs are present within oral tissues for instance as periodontal ligament stem cells (PDLSCs) (Widera et al., 2007; Huang et al., 2009; Kawanabe et al., 2010), dental pulp stem cells (DPSCs) (Gronthos et al., 2000; Stevens et al., 2008; reviewed in Sloan and Waddington, 2009; Kaltschmidt et al., 2012), human oral mucosa stem cells (hOMS) (Marynka-Kalmani et al., 2010) and progenitor cells in the lamina propria of the oral mucosa (OMLP-PC) (Davies et al., 2010), or oral stromal stem cells (Boddupally et al., 2016). Although expression of neural crest-associated markers in PDLSCs seems to be heterogeneous in dependence to the culture system, several studies reported successful differentiation into a range of ectodermal and mesenchymal derivates (Widera et al., 2007; Huang et al., 2009). However, single cell-derived PDLSC-clones were demonstrated to differ in their differentiation capability ranging from clones with multilineage potential to sole osteoprogenitors (Singhatanadgit et al., 2009).

Next to periodontal ligament, dental tissues particularly including the dental pulp are developmentally derived from the neural crest (Chai et al., 2000). Human DPSCs were reported to be positive for the NCSC-marker Nestin (Arthur et al., 2008) and to form dentin and dental pulp tissue *in vivo*, but are also able to give rise to osteoblasts, chondrocytes, adipocytes and even to NC-related melanocytes and functionally active neurons (Gronthos et al., 2000; Arthur et al., 2008; Stevens et al., 2008; Jiang et al., 2019). In accordance with our own observations in human ITSCs, single cell-derived clonal cultures of murine DPSCs revealed highly heterogeneous gene expression levels of Nestin between individual stem cell clones. Notably, DPSCs showing high Nestin expression differentiated into neuron-like or oligodendrocyte-like derivates, while Nestin^low^-DPSCs lacked this capacity (Young et al., 2016). In line with these findings, Kobayashi et al. (2020) recently characterized differences in proliferation, differentiation potential and gene expression between fifty human dental pulp stem cell clones. Although the characteristic DPSC-markers STRO-1 and CD146 were co-expressed in nearly all DPSC clones, individual DPSCs showed great differences in their differentiation capacities into odontogenic, adipogenic, and chondrogenic derivates (Kobayashi et al., 2020). Likewise, Jiang and colleagues observed successful differentiation of a human DPSC clone into the osteogenic, adipogenic, and chondrogenic lineage, while two distinct clones from the same parental population only revealed osteogenic differentiation capacity (Jiang et al., 2019). In this line, Gronthos et al. (2000) reported *in vivo* generation of ectopic dentin by nine out of twelve individual single-colony-derived DPSCs. On the contrary, three out of twelve clonally grown DPSCs revealed only a limited capability of forming dentin, indicating great heterogeneity between individual DPSCs regarding their differentiation capability (Gronthos et al., 2000). On technical level, heterogeneity of human DPSCs was recently shown to be assessable by single-cell Raman spectroscopy (SCRM). Alraies et al. (2019) utilized SCRM to discriminate subpopulations of DPSCs from human third molars and established SCRM-fingerprints defining highly proliferative/multipotent and low proliferative/unipotent DPSCs. This highly promising technique may enable selective non-invasive screening of DPSCs in the future and further validates the heterogeneity between individual NC-derived DPSCs. Determining the molecular basis for the commonly observed differences in proliferative behavior, Alraies and colleagues identified a relation between proliferative heterogeneity of DPSCs and differences in telomere lengths and CD271 expression, suggesting variations in human dental pulp stem cell aging (Alraies et al., 2017).

## Heterogeneity of NCSCs in the Carotid Body and Cornea of the Eye

Located in the bifurcation of the carotid artery, the carotid body (CB) is the main arterial chemoreceptor sensing oxygen (Lopez-Barneo et al., 2001) and was reported to contain cells of neural crest origin in Pearse et al. (1973). NCSCs were discovered to be present in the adult rat CB by Pardal et al. (2007) and revealed the capacity to form spheres and give rise to dopaminergic neurons and mesenchymal cells *in vitro*. Strikingly, the authors demonstrated CB-NCSCs to remain in a quiescent glia-like cell state until activation by hypoxia, which in turn resulted in a phenotype switch toward Nestin-positive NCSCs undergoing neurogenesis *in vivo* (Pardal et al., 2007; Platero-Luengo et al., 2014). Next to contributing to neurogenesis in the CB during persistent hypoxia, the group around López-Barneo further reported murine CB-NCSCs to efficiently give rise to endothelial cells directly contributing to hypoxia-induced angiogenesis (Annese et al., 2017). These promising data emphasize the enormous plasticity of adult NCSCs commonly described to undergo mesenchymal or ectodermal rather than endothelial differentiation. Although investigations of their plasticity still remain to be investigated in human CB-NCSCs, the data provided by the López-Barneo group further indicate a niche-dependent heterogeneity of NCSCs in terms of their differentiation potential, which is in accordance to our own very recent findings (Höving et al., 2020b).

Next to the carotid body, the oral and nasal cavity as well as the olfactory bulb, the human eye likewise harbors craniofacial NCSCs. In this regard, Yoshida et al. (2006) demonstrated the presence of NCSCs positive for Nestin, SOX9, TWIST, SLUG, and SNAIL in the adult murine cornea. Such NC-derived corneal precursors (COPs) revealed the capacity to differentiate into keratocytes, adipocytes and chondrocytes, while only showing a minor potential to undergo neuronal differentiation (Yoshida et al., 2006). NCSCs expressing Nestin, SOX9, SNAIL, SLUG, and TWIST were further reported to be located in the murine corneal limbus (Brandl et al., 2009). ABCB5-positive human limbal stem cells (LSCs) capable of restoring the corneal epithelium upon transplantation into LSC-deficient mice (Ksander et al., 2014) are likewise suggested to be of neural crest origin (reviewed in Gonzalez et al., 2018). However, although the limbal niche is commonly known to harbor NC-derived limbal stromal fibroblasts (Gage et al., 2005) and melanocytes, its complexity and heterogeneity challenges the determination of the identity and developmental origin of LSCs (reviewed in Gonzalez et al., 2018). In addition, NCSC-markers were observed to be expressed in neural crest-derived progenitors isolated from the adult human corneal endothelium, which revealed the capacity to undergo differentiation into the neuronal lineage and the corneal endothelium itself (Katikireddy et al., 2016). As discussed above, the complexity of this already highly heterogeneous microenvironment accompanied by the lack of single cell data regarding potential human corneal/limbal NCSCs so far likewise challenges the assessment of NCSC-heterogeneity in the limbal and corneal niche.

## Heterogeneous Expression Patterns and Plasticity of NCSCs in the Craniofacial and Trunk Skin

The human craniofacial skin is known to harbor two distinct populations of NCSCs, namely skin-derived precursors (SKPs) (Toma et al., 2001; Fernandes et al., 2004) and epidermal neural crest stem cells (EPI-NCSCs) located within the bulge of hair follicles (Sieber-Blum et al., 2004). Next to their presence in the craniofacial region, EPI-NCSCs were also identified within the trunk region of the human body (Clewes et al., 2011). While SKPs were reported to express Nestin, SNAIL, SLUG, TWIST and SOX9 (Toma et al., 2001; Fernandes et al., 2004), EPI-NCSCs were shown to lack expression of Slug, Snail and Twist. On the contrary, EPI-NCSCs were shown to display expression of Msx2 and SOX10 (Sieber-Blum et al., 2004; Hu et al., 2006), emphasizing the transcriptional heterogeneity of NCSCs even between bulk populations located close to each other. Interestingly, an assessment of the adult murine epidermis on single level by Joost et al. (2016) suggested that self-renewing cells in the adult hair follicle lack a distinct stemness gene expression signature. On the contrary, self-renewing cells shared a common basal gene expression signature accompanied by characteristic spatial signatures segregating the cell populations in relation to their location during tissue homeostasis (Joost et al., 2016). Two years later, Joost and colleagues analyzed hair follicle bulge stem cells positive for LGR5, a marker for NCSCs in the oral cavity (Boddupally et al., 2016), on single cell level to asses transcriptional adaptations during wound healing in mice (Joost et al., 2018). Interestingly, LGR5-positive hair follicle bulge stem cells gradually activated an interfollicular epidermis stem cell-like gene expression signature even before migrating out of the bulge toward the lesion. These findings suggest a great cellular plasticity of single LGR5-positive hair follicle bulge stem cells allowing rapid transcriptional adaptations during wound healing (Joost et al., 2018). Accordingly, EPI-NCSCs were also described to show great cellular plasticity (Sieber-Blum et al., 2004; Hu et al., 2006; Hu et al., 2010) but no reports addressing their intrapopulational heterogeneity are available so far. Single SKPs from human foreskin were shown to give rise to mesenchymal and ectodermal cell types like neurons or smooth muscle cells (Toma et al., 2001), although Dai et al. (2018) suggested SKP-spheres to contain a heterogeneous mixture of stem and progenitor cells. These suggestions are in accordance with our own observations regarding the different ratios of single ITSC-derived ectodermal to mesenchymal progeny (Greiner et al., 2011; Hauser et al., 2012; see also section “Fate Specifications and Heterogeneity of NCSCs From the Nasal Cavity and Olfactory Bulb” and Figure 3C) as well as the heterogeneous expression of NC-markers in ITSCs (Figure 4).

## Heterogeneity of Trunk NCSCs in the Dorsal Root Ganglia, Sciatic Nerve, Bone Marrow, Heart, and Gut

Trunk NCSC populations can be found in the gut, bone marrow, sciatic nerve, dorsal root ganglia and the heart (Kruger et al., 2002; Mosher et al., 2007; Nagoshi et al., 2008; Coste et al., 2017; Höving et al., 2020a, b). For instance, clonally grown colonies of p75^+^ NCSC from the postnatal rat gut showed multilineage potential containing neurons, glia, and myofibroblasts (Kruger et al., 2002). Nagoshi et al. (2008) compared mouse NCSCs from bone marrow, dorsal root ganglia and whisker pad and detected tissue source-dependent differentiation capacities. Compared with whisker pad- and bone marrow-derived NCSCs, dorsal root ganglia-derived NCSCs showed a higher degree of sphere formation and increased expression of p75, Nestin, SOX10, and Musashi1 accompanied by increased differentiation capacity into neurons, glia and myofibroblasts (Nagoshi et al., 2008). Morrison and colleagues isolated p75^+^ NCSC from the rat fetal sciatic nerve. These cells possessed the capacity to expand on clonal level and to differentiate into neurons, Schwann cells and myofibroblasts. Moreover, the neurotrophin receptor p75 and the peripheral myelin protein P0 were utilized to isolate subpopulations of cells with varying developmental potentials. Notably, high expression of p75 compared with a lack of P0 was accompanied by a high frequency (60%) of multipotent clones giving rise to neurons, Schwann cells and myofibroblasts while p75^+^P0^+^ cells showed only a frequency of 28% of multipotent clones. In addition, clonal cultured p75- cells were only able to differentiate into myofibroblasts independent of the expression of P0 (Morrison et al., 1999). These results suggest p75 to be one of the major markers for self-renewing, multipotent NCSCs. Accordingly, we detected higher proliferation rates in p75^+^ subpopulations of ITSCs compared to p75^–^ ITSCs (Hauser et al., 2012). Moreover, a recent study from Coste and colleagues identified a Nestin^+^/SOX9^+^/TWIST^+^ NCSC population in the human bone marrow with the ability to follow neural crest migration pathways after transplantation into chick embryos (Coste et al., 2017). Populations of NCSCs in the adult heart were firstly described in mice and zebrafish (Tomita et al., 2005; El-Helou et al., 2008; Hatzistergos et al., 2015; Leinonen et al., 2016; Meus et al., 2017; Tang et al., 2019). Recently, we identified a Nestin^+^/cKit^–^ stem cell population in the human heart (human cardiac stem cells, hCSCs) with NCSC properties, giving rise to neuron-like cells, adipocytes and cardiomyocytes (Höving et al., 2020a, b). However, in a direct comparison with ITSCs as cranial NC-derivates we observed a relatively minor potential of hCSCs to undergo neuronal differentiation (Höving et al., 2020b). A general difference between NCSC populations from different tissues and niches was also reviewed by Shakhova and Sommer (2008). In general, trunk NCSCs from dorsal root ganglia, sciatic nerve and gut seem to possess a higher capacity for ectodermal differentiation, particularly into neurons and glia compared to their mesenchymal differentiation potential into osteogenic or adipogenic cell types (reviewed in Shakhova and Sommer, 2008). Recently, Groeneveldt et al. (2020) performed a direct comparison of human periosteum-derived cells (hPDCs) from the tibia as a mesoderm-derived tissue with hPDCs from maxilla and mandible as examples of cranial neural crest-derived cells. While all cell populations exhibited similar differentiation capacities into chondrogenic, adipogenic and osteogenic derivates and proliferation rates *in vitro*, global gene expression analysis showed a higher amount of differentially expressed genes (DEG) between hPDCs from the tibia and each of the craniofacial hPDC populations than between hPDCs from the maxilla and mandible. In addition, the expression of HOX family members was upregulated in tibia-hPDCs compared to maxilla- or mandible-hPDCs. After implantation in nude mice, tibia- and mandibular- but not maxilla-hPDCs participated to bone formation. These different properties in differentiation potentials *in vivo* may be associated to the differential expression of genes from the HOX and DLX family (Groeneveldt et al., 2020). Likewise, Leucht et al. (2008) detected differences in the contribution to bone repair mechanisms between murine neural crest-derived skeletal stem cells and mesoderm-derived cells, where the expression of Hoxa11 plays a crucial role. In this regard, transplantation experiments in quail-chick chimeras showed that HOX-negative embryonic neural crest cells are able to adopt the HOX status of a HOX-positive environment. Vice versa, transplanted HOX-positive NCCs did not lose their HOX-status in a new HOX-negative environment (Grapin-Botton et al., 1995; Couly et al., 1998). The here discussed findings emphasize the relevance of HOX-activation in neural crest development and indicate its role in regulating adult NCSC-heterogeneity. Heterogeneous marker expression or differentiation potentials between NCSC-populations derived from different embryological origins are further discussed in section “Potential Origins of NCSC-Heterogeneity.” On the contrary, NCSCs from bone marrow were reported to efficiently give rise to neuronal and mesenchymal cell types. Notably, the embryonic counterparts of some of these adult NCSCs populations were also reported to be heterogeneous in response to their microenvironment *in vivo* and *in vitro* (Bixby et al., 2002; Wong et al., 2006; see also Shakhova and Sommer, 2008 for review). For instance, E14 rat NCSCs from sciatic nerve were shown to be more responsive to gliogenic factors, while NCSCs from the gut revealed a greater responsiveness to neurogenic factors (Bixby et al., 2002). In addition, transplantation of either NCSC from the gut or from the sciatic nerve into chick embryos showed population-specific differences. Here, gut NCSCs efficiently migrated and formed enteric neurons in the developing gut, but NCSCs from the sciatic nerve did not reveal this capacity (Mosher et al., 2007). Although these studies describe a high degree of heterogeneity between different NCSC-populations, to the best of our knowledge, studies addressing the issue of cellular heterogeneity within these adult NCSC-populations in the human organism still remain elusive.

## Molecular Regulators Defining Stemness and Fate Choices of Ncscs

**FIGURE 5:**
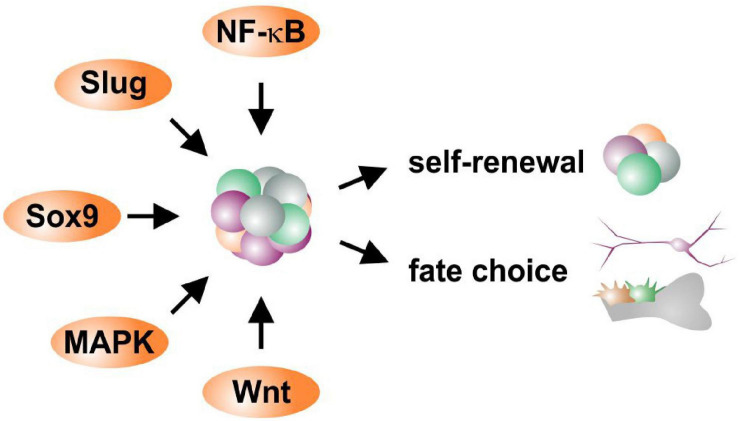
Schematic view of potential molecular regulators defining self-renewal and fate choices of NCSCs.

Heterogeneity within and between the NCSC-populations discussed above is directly related to the activity of potential molecular regulators defining their stemness and differentiation behavior. According to the role of EMT being a prerequisite for migration of embryonic neural crest cells out of their niche, common EMT-drivers like SOX9, SOX10, TWIST, SLUG and SNAIL are still present in adult NCSCs (see also section “Adult Neural Crest-Derived Stem Cells—From Individual Developmental Drivers to Mediators of Regeneration During Adulthood”). Notably, activation of EMT is closely associated to stem cell properties, with EMT transcription factors regulating stemness (Lamouille et al., 2014; Nieto et al., 2016). EMT drivers were also described to regulate each other with SNAIL inducing upregulation of TWIST (Casas et al., 2011; Dave et al., 2011), which in turn both positively regulate SLUG (Boutet et al., 2006; Casas et al., 2011). We suggest this regulatory network of EMT-drivers to be vital for maintaining NCSC-stemness (Figure 5). In this line, SOX10was demonstrated to be necessary for maintaining multipotency and inhibiting neuronal differentiation of neural crest cells (Kim et al., 2003). The EMT-transcription factor SLUG is also particularly noticed to regulate and determine stemness states of adult stem cells (Tang et al., 2016). Interestingly, SLUG and SOX9were also reported to cooperatively determine the stemness state of human breast cancer stem cells (Guo et al., 2012). Our very recent observations show a great heterogeneity in expression and protein amounts of SLUG between individual ITSCs (Figure 4), suggesting a direct association to the observed differences in differentiation potentials between single ITSCs (see also section “Fate Specifications and Heterogeneity of NCSCs From the Nasal Cavity and Olfactory Bulb,” Figure 3C). Interestingly, canonical Wnt-signaling, which is closely linked to EMT, was described as a major regulator promoting neuronal fate choices of HBCs, thus driving the route of HBCs from quiescence to neuronal differentiation (Fletcher et al., 2017). Accordingly, canonical Wnt-signaling is commonly noticed to maintain the stemness of neural crest stem cells and ASCs and guide ASC-differentiation in dependence to the environmental context (Figure 5; Kleber et al., 2005; Ring et al., 2014).

Next to EMT-drivers regulating each other, the transcription factor NF-κB was described to influence their expression and activity. Here, the regulation of SOX9, SLUG and TWIST was reported to be mediated by NF-κB in cancer stem cells and during early vertebrate development (Zhang et al., 2006; Sun et al., 2013). In particular, binding of NF-κB to κB binding sites present in their promoters was shown to lead to increased expression of SLUG, SOX9 and TWIST in breast and pancreatic cancer, in turn enabling EMT (Li et al., 2012; Sun et al., 2013; Pires et al., 2017). With SLUG, SOX9, and TWIST as well as NF-κB being commonly present in NCSCs (Toma et al., 2001; Fernandes et al., 2004; Hauser et al., 2012; Müller et al., 2016; Ruiz-Perera et al., 2018), we suggest a similar network being present in adult NCSCs directly influencing fate decisions (Figure 5). Accordingly, we recently demonstrated a fate shift of ITSCs from the neuronal to oligodendroglial lineage by inhibition of NF-κB c-Rel (Ruiz-Perera et al., 2020). Interestingly, we very recently identified p38-MAPK-signaling to be crucial for protection of human cardiac NCSCs from senescence and promoting their proliferation (Höving et al., 2020a), suggesting an additional regulatory role in NCSCs (Figure 5). In addition to EMT-drivers and NF-κB, the expression of the NCSC-marker Nestin is also directly associated with the differentiation capacity of NCSCs. Young et al. (2016) demonstrated a highly heterogeneous expression of Nestin between individual DPSCs clones, with only Nestin^high^ DPSCs being capable of neuronal and oligodendrocyte differentiation (see also section “Heterogeneity of NCSCs in the Oral Cavity”). We likewise observed differences in Nestin expression between single ITSCs, suggesting a linkage to differences in differentiation potential (see also section “Fate Specifications and Heterogeneity of NCSCs From the Nasal Cavity and Olfactory Bulb”, Figure 3C, 4A). An additional potential marker for multipotent self-renewing subpopulations could be the neurotrophin receptor p75. As discussed above (see section “Heterogeneity of Trunk NCSCs in the Dorsal Root Ganglia, Sciatic Nerve, Bone Marrow, Heart, and Gut”), p75^+^ populations of rat sciatic nerve-derived NCSCs were able to differentiate into neurons, Schwann cells and myofibroblasts while p75^–^ cells were only able to differentiate into myofibroblasts (Morrison et al., 1999). In our lab, p75^+^ adult ITSCs exhibited increased proliferation rates compared to their p75^–^ counterparts (Hauser et al., 2012). In summary, an interplay between major EMT-drivers like SOX9 or SLUG with NF-κB seems likely to orchestrate the stemness state of adult NCSCs, although the exact regulatory mechanisms currently remain unknown (Figure 5). Canonical Wnt- and MAPK-signaling seem to play an additional role in mediating proliferation and fate decisions of NCSCs (Figure 5).

## Potential Origins of NCSC-Heterogeneity

**FIGURE 6:**
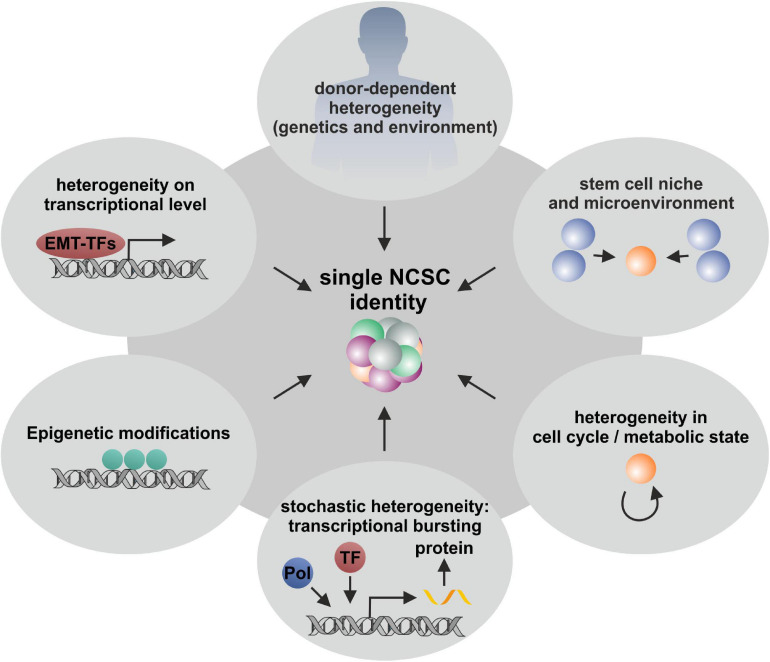
Schematic illustration of various extrinsic and intrinsic factors influencing NCSC heterogeneity. Single stem cell heterogeneity is influence by diverse variables including donor-to-donor variations, the stem cell niche, epigenetic modifications, stochasticity of mRNA and protein production and random segregation during cell division. All these aspects are involved in stem cell heterogeneity and are crucial for the development and the existence of multicellular organisms.

Cellular heterogeneity is known to be a general feature of multicellular organisms. Despite donor-to-donor heterogeneity (Hauser et al., 2012; Belderbos et al., 2020; Figure 6), especially transcriptional lineage segregation during human embryonic development as well as the requirement of tissue-specific functionality in adulthood are reasons for distinct gene expression profiles (Xue et al., 2013; Yan et al., 2013). This kind of lineage segregation also occurs in the development of different subpopulations deriving from neural crest stem cells, which give rise to a broad variety of ectodermal and mesenchymal cell types during embryogenesis (Dupin and Sommer, 2012; Kaltschmidt et al., 2012; Etchevers et al., 2019). In accordance to their specific position on the anteroposterior axis, differentiation potential as well as migration behavior is coordinated resulting in cranial, vagal (including cardiac), trunk and sacral NC cells (Rothstein et al., 2018; Rocha et al., 2020; see also section “Adult Neural Crest-Derived Stem Cells—From Individual Developmental Drivers to Mediators of Regeneration During Adulthood”). Such developmental-based heterogeneity results in tissue-specific gene expressions, which were recently investigated by Han and colleagues using scRNA-seq to determine the cell-type composition of all major human organs, leading to the construction of a scheme for the human cell landscape. Additionally, the authors commented on discrepancies between differentiated cell types and stem cells concerning solidity of gene expression, as stem and progenitor cells revealed more instable transcription profiles (Han et al., 2020). These observations are in accordance with reported heterogeneity concerning differentiation potentials as well as expression profiles of NCSCs deriving from different tissues (Höving et al., 2020b; Figure 6). Such a niche-dependent heterogeneity is most likely driven by different extrinsic stimuli based on variations within the microenvironments harboring stem cells (Trentin, 1971; Metcalf, 1998). An influence of extrinsic factors on heterogeneity may be also reflected by different culture conditions of NCSCs, as serum-free culture was shown to favor neuronal fate decisions in comparison to serum-containing culturing, which was shown to support proliferation (Ziller et al., 1983). This was even reported for clonally grown NCSCs, where fate choices were influenced by the application of specific growth factors (Sieber-Blum and Cohen, 1980). Calloni and colleagues isolated embryonic cranial NCCs from the quail mesenchephalon and could show that treatment with the morphogen Sonic Hedgehog (Shh) increased the number of multipotent subclones with the capacity to differentiate into glia, neurons, melanocytes, myofibroblasts and chondrocytes (GNMFC-progenitors) while untreated clones where more restricted to neural progenitors differentiating into neurons, glia and melanocytes (GNM-progenitors) (Calloni et al., 2007, 2009). Likewise, da Costa and colleagues detected in clonally grown quail embryonic mesencephalic NCCs increased developmental potential into glial cells, neurons, melanocytes, smooth muscle cells, chondrocytes, and adipocytes after the simultaneous application of FGF8 and Shh (da Costa et al., 2018). Moreover, Dupin and colleagues reviewed the differentiation capacities of embryonic NCCs from the cephalic and trunk regions of the neural crest, discussing that trunk NCCs possess an *in vivo* differentiation potential into the neural, glial and melanocytic lineage which can be extended *in vitro* to mesenchymal cell types like connective, osteogenic, adipogenic and skeletogenic cells with Shh playing a central role (Dupin et al., 2018). Nevertheless, stem cell heterogeneity within purified populations and even in clonally derived cell lines may not only be regulated by extrinsic, but also intrinsic factors. As discussed above, clonal cultures of oral cavity-derived NCSCs revealed specific variations in their differentiation potential under uniform culture conditions (Jiang et al., 2019; Kobayashi et al., 2020). This was also reported for ITSC clones, which gave rise to different ratios of ectodermal to mesodermal progeny upon differentiation (see also section “Fate Specifications and Heterogeneity of NCSCs From the Nasal Cavity and Olfactory Bulb”). Notably, ITSCs were precultivated using animal serum-free 3D cultivation methods assuring genetic stability and stemness including mesenchymal and ectodermal differentiation *in vitro* (Greiner et al., 2011; Hauser et al., 2012). Accordingly, Kerosuo et al. (2015) postulated conserved multipotency of clonal NCSCs via long term culturing as so-called “crestospheres,” although the authors observed dynamic heterogeneity in the expression of neural crest markers within clonally grown spheres. Nevertheless, this 3D culture method was shown to maintain NCSCs self-renewal and multipotency for weeks avoiding spontaneous differentiation (Kerosuo et al., 2015) and substantially reducing heterogeneity. However, the here discussed observations emphasize the necessity to critically monitor cultivation conditions as a potential source of spontaneous differentiation and artificial heterogeneity.

Further intrinsic factors driving heterogeneity of stem cells may include differences in gene expression upon transcriptional regulation (see also section “Molecular Regulators Defining Stemness and Fate Choices of NCSCs”), cell cycle state (Kowalczyk et al., 2015; Tsang et al., 2015), epigenetic heterogeneity based on differences in chromatin states (Yu et al., 2017; reviewed in Carter and Zhao, 2020) as well as stochastic fluctuations of the mechanisms underlying mRNA and protein production (Raj et al., 2008; see Figure 6 for overview). Such stochastic fluctuations have their origin in random segregation at the cell division stage (Huh and Paulsson, 2011) and are essential for spontaneous generation of complex patterns and thus are crucial driver of evolution and selection (Fraser et al., 2004). In this line, Wu and Tzanakakis (2012) analyzed the heterogeneous expression of the pluripotency marker Nanog in genetically identical human ESCs and linked the observed heterogeneity to stochastic partitioning at division and transcriptional noise. Intrinsic stochastic heterogeneity of transcription is emphasized to rely on a process named transcriptional bursting, which describes the stochastic activation and inactivation of promoters (Raj et al., 2006; Fukaya et al., 2016). Recently, Ochiai et al. (2020) used scRNA-seq to investigate the reasons for this process in murine ESCs and reported kinetic properties of transcriptional bursting to be influenced by multiple promoter-binding proteins, such as transcription elongation factors. Although the influence of such stochastic events on NCSC heterogeneity cannot be completely excluded, the here discussed observations emphasize the presence of distinct NCSC-stemness states being based on differences in gene expression driven by molecular regulators like EMT-drivers as well as NF-κB, canonical Wnt-, and MAPK-signaling (see also section “Molecular Regulators Defining Stemness and Fate Choices of NCSCs” Figure 6). In this regard, the potential further influence of cell cycle states and epigenetic regulation need to be assessed in future studies. The findings summarized here further strongly suggest heterogeneity of NCSCs to be partly determined by their niche of origin including the external and internal factors defined by the microenvironment as discussed above.

## Summary and Outlook: NCSC-Identities Between Heterogeneous Differentiation Potential and Common Transcriptional Signatures

In summary, the present review emphasizes the great heterogeneity of craniofacial and trunk NCSC-populations. NCSC-heterogeneity was reported to be present on multiple levels particularly including the donor, the sex of the donor, the cell population and the single stem cell. On donor level, variations between donor-to-donor were described in the amount of NCSCs in their niche as well as sex-specific differences in behavior during differentiation. Interpopulational differences further substantially contribute to the heterogeneity between NCSCs observed in differentiation potential with particularly regard to their niche of origin. On single cell level, differences in expression signatures of NCSCs were further directly linked to individual fate decisions *in vitro* and *in vivo*. With regard to these diverse levels of heterogeneity, even among clonally grown NCSCs, there is no overall ideal source of NCSCs or culture condition to overcome single cell heterogeneity so far. Nevertheless, clonal 3D-culture methods, including sphere cultures (Kerosuo et al., 2015) or matrices (Greiner et al., 2011; Hauser et al., 2012) are increasingly noticed to reduce spontaneous differentiation, thus at least overcoming one parameter of extrinsic heterogeneity. Despite the broad heterogeneity of NCSCs, we suggest that the global presence of EMT-associated transcription factors like SLUG, SOX9, or SOX10 is not only prerequisite for NCSC-identity but orchestrates their stemness state in co-regulation with NF-κB, canonical Wnt-, and MAPK-signaling. In addition, the neurotrophin receptor p75 may label subpopulations of NCSCs with enhanced proliferation rates and differentiation capacities (Morrison et al., 1999; Hauser et al., 2012). The observed heterogeneity in the expression of these NCSC markers may thus directly contribute to heterogeneous stemness states but also further emphasizes the need for a definite set of markers verifying NCSC-identity.

As a future perspective, comparisons between NCSC-populations of different niches may shed light on the basis of heterogeneity observed in their differentiation potential. In this regard, we recently demonstrated high similarities between the transcriptomes of craniofacial and cardiac human NCSCs, despite the developmental differences between embryonic cranial and vagal NC cells. On the contrary, the assessed global gene expression signatures likewise reflected differences between the adult NCSC-populations with regard to their particular niche (Höving et al., 2020b). In this line, we also observed differences in their differentiation potential likewise reflecting their origin in the craniofacial region or the adult heart (Höving et al., 2020b). Next to comparing NCSC-populations, single cell analysis will serve as a powerful tool to assess individual heterogeneities between NCSCs and broaden our understanding of NCSC fate choices in tissue regeneration. However, single cell heterogeneity will be an additional challenge for therapeutic applications of NCSCs, as one prerequisite of cell therapy is consistency of cell populations as medical product to render unvarying clinical results. Overcoming heterogeneity within an even clonally grown cell population will be nearly impossible, thus cellular products for therapies have to be characterized regarding specific phenotype and molecular mechanisms essential for the treatment of the respective disease. Consequently, heterogeneity of NCSCs increases the complexity in developing cell-based therapeutics and single cell analysis may provide new insights in the consequences of cellular heterogeneity in clinical applications. In this line, the findings discussed here emphasize the assessment of heterogeneity of NCSCs between donors, cell populations and single stem cells to be vital for understanding their roles in tissue homeostasis and improving their applicability in regenerative medicine.

## Author Contributions

JG provided the conception and design of the manuscript. AH, BW, and JG wrote the manuscript. BK, CKa, and CKn revised the manuscript critically for important intellectual content and provided funding. All authors read and approved the submitted version of the manuscript.

## Conflict of Interest

The authors declare that the research was conducted in the absence of any commercial or financial relationships that could be construed as a potential conflict of interest.
